# Evaluation of a Self-Test Technology for Chlamydia and Gonorrhea Testing: Observational Before-and-After Study

**DOI:** 10.2196/99694

**Published:** 2026-09-15

**Authors:** Bettina Trettin, Ida Wagner Josefsson, Nadja Trier Munk, Tine Vestergaard, Tue Kjølhede, Mette Maria Skjøth

**Affiliations:** 1 Department of Dermatology and Allergy Centre, Odense University Hospital, J.B. Winsløws Vej 4, Odense, South Denmark, 5000, Denmark, 45 60494279; 2 Clinical Institute, Health Sciences, University of Southern Denmark, Odense, South Denmark, Denmark; 3 Centre for Innovative Medical Technology, Odense University Hospital, Odense, South Denmark, Denmark

**Keywords:** sexually transmitted infections, chlamydia, self-testing, evaluation, health technology

## Abstract

**Background:**

Chlamydia remains highly prevalent, and the prevalence of gonorrhea is increasing. Declining testing rates alongside rising positivity in Denmark highlights the need for accessible testing solutions. Self-test technologies (STTs) may reduce stigma and improve testing uptake.

**Objective:**

This study aimed to examine the effects of implementing an STT on activity-related outcomes, including test volume, testing frequency, and positivity rates for sexually transmitted infections (chlamydia and gonorrhea) at a sexual health clinic.

**Methods:**

A retrospective before-and-after study evaluated the implementation of an STT located in a sexual health clinic at a university hospital over the period of 2022 to 2025. The evaluation consisted of 3 phases: preimplementation phase (phase 0), self-test with booking (phase 1), and drop-in self-test (phase 2). Outcomes included testing volume, frequency, positivity rates, staff resource use, and costs, assessed using descriptive statistics and chi-square tests.

**Results:**

The monthly tests increased from a mean of 163 (SD 51) before implementation to 246 (SD 42) and 283 (SD 78) in phases 1 and 2, respectively. The proportion of patients tested multiple times increased significantly (*P*=.001). Positivity rates declined from 15.7% (180/1144) to approximately between 12% (652/5417) and 12.6% (464/3689; *P*=.003). Weekly staff time decreased from 102 to 63.75 hours, and annual personnel costs declined by 37%. Despite increased testing volume, the cost per test decreased from DKK 1179 (US $185) to DKK 501 (US $79).

**Conclusions:**

Implementation of an STT increased testing activity and efficiency while reducing costs and staff resource use, suggesting that self-testing is a scalable and resource-efficient strategy for sexually transmitted infection diagnostics in clinical settings.

## Introduction

Chlamydia remains the most prevalent bacterial sexually transmitted infection (STI) worldwide, whereas gonorrhea is the second most common, and its incidence is increasing. Both infections may lead to pelvic inflammatory disease and serious reproductive complications, including ectopic pregnancy and infertility, if left untreated [1-3]. In Denmark, testing rates are declining, whereas the positivity rate is on the rise [4], which emphasizes the need to increase testing uptake and make an effort to reduce transmission. In Denmark, most patients, who are mainly young sexually active people, are tested at their general practitioner. Additionally, there are 6 sexual health clinics offering STI testing. Both options are free of charge. Furthermore, internet-ordered postal home-sampling kits exist, but patients have to wait for days, sometimes weeks, for a result and, if it is positive, have to attend a face-to-face consultation at their general practitioner [5]. This may be a problem as STI testing is associated with embarrassment, stigma, and shame [6-8], and thus, patients request discreet and anonymous testing, preferably without any face-to-face contact with a health care professional.

There is only limited evidence on how acceptable home testing is. Internet-based self-sampling may not be the preferred testing by users, and there seems to be a need for alternative testing strategies [5]. However, it prevents cases of pelvic inflammatory disease and is cost-effective compared to traditional clinic-based screening [9]. To address feelings of embarrassment and stigma and increase testing, a self-test technology (STT) that allows patients to be tested at a sexual health clinic through self-collected sampling without a face-to-face consultation was developed and tested in a previous study [10]. Thus, patients could attend the clinic as a drop-in at any time during its opening hours. On arrival, they had to scan their social security card at the STT, which verified their identity and linked the test to their electronic medical record. Instructions for self-collection were provided through written guides and video. The STT was co-designed and developed with end users, and an evaluation of its use consisting of qualitative research methods showed that it minimized feelings of shame and awkwardness [10]. The STT was pilot-tested and implemented into daily operation. Using the STT at a sexual health clinic located at a hospital empowered patients as it gave them control to make choices about when to take a test [10] and the opportunity to be tested easily and quickly and obtain a result within 1 or 2 days. In addition to minimized feelings of shame and awkwardness, patients experienced it as an easy solution that both saved time and allowed for flexibility to plan their visits. This resulted in greater confidence and feelings of self-determination and, therefore, has the potential to increase testing uptake [10]. Thus, self-testing may facilitate earlier detection and treatment, thereby reducing onward transmissions and long-term complications such as pelvic inflammatory disease. While improving access to testing is a key objective of STTs, easier access may also have unintended consequences. Reduced barriers to testing could encourage individuals to undergo testing more frequently than clinically indicated, resulting in a higher proportion of negative tests and increased repeated testing among the same individuals. Such changes could have implications for health care resource use and cost-effectiveness. Therefore, in addition to evaluating whether implementation of the STT increased testing uptake, it was important to examine whether improved accessibility was associated with changes in testing frequency and positivity rates that might indicate potential overuse.

The STT was located in a sexual health clinic in a discrete setting at a university hospital and was fully implemented in clinical practice, with over 3400 users per year. Although the qualitative evaluation indicated that the STT has the potential to increase testing rates, it remains important to examine the clinical, organizational, and economic effects of its implementation. Thus, the aim of this study was to examine the effects of implementing an STT on activity-related outcomes, including test volume, testing frequency, and positivity rates for STIs (chlamydia and gonorrhea) at a sexual health clinic. In addition, the consequences for staff resource use and overall economic implications were assessed.

## Methods

### Study Design and Setting

This retrospective observational before-and-after study was conducted at the Department of Dermatology and Allergy Centre, Odense University Hospital, Denmark. The aim was to evaluate the implementation of the STT for chlamydia and gonorrhea, focusing on its impact on test activity (volume, frequency, and positivity rates), staff resource use, and associated costs.

### Implementation Phases

#### Overview

Data were analyzed across 3 distinct periods:

- Phase 0 (preimplementation phase): February 19, 2022, to October 23, 2022
- Phase 1 (self-test with booking): October 24, 2022, to September 1, 2024
- Phase 2 (drop-in self-test): September 2, 2024, to October 20, 2025

These phases were defined according to the introduction and operational workflow of the STT, which allowed patients to get tested either after a scheduled booking or as drop-in without direct health care professional involvement

#### Phase 0: Preimplementation Phase

Before the implementation of the STT, all tests were performed at face-to-face consultations held by either 2 nurses or a nurse and a physician. All STI tests are performed using nucleic acid amplification tests (NAATs), which are considered a “gold standard” and used in laboratory diagnosis. Patient-collected samples for chlamydia and gonorrhea using NAATs are feasible and acceptable, with very high agreement between health care professional-collected and self-collected tests [11].

#### Phase 1: Self-Test With Booking

To use the STT, patients had to call a nurse trained in venerology, who conducted a brief interview and entered patient information into the electronic medical record. Patients were then given access to the STT using their personal identification number. Men were instructed to self-collect a urine sample, and women were instructed to self-collect a vaginal swab. Patients had a window of 14 days to use the STT during clinic drop-in hours and were informed that positive results would be sent electronically to their secure digital mailbox, whereas negative results could be checked via the Danish national portal for patient communication[12].

#### Phase 2: Drop-In Self-Test

**Figure 1.**
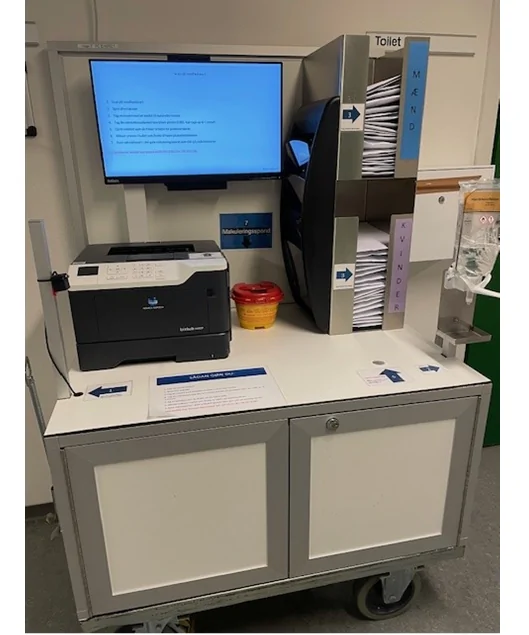
The self-test technology.

The STT was further developed so that patients could access it without any contact with a health care professional and, consequently, without the need for prior booking (Figure 1). Textbox 1 provides further details on the process.

Textbox 1. **Details on the process of self-testing with drop-ins.**

- Upon arrival, patients scanned their national health insurance card at the self-test technology (STT), which verified their identity and linked the test to their electronic health record.
- Patients then collected a self-sampling kit from the STT. Instructions for self-sampling were provided through both written guidance and instructional videos.
- The sample was collected by the patient in a nearby restroom and subsequently deposited into a locked compartment in the self-testing station.
- Samples were collected daily and transported to the laboratory for analysis.
- Patients were informed that positive test results would be delivered electronically to their secure digital mailbox, whereas negative results could be accessed through the Danish national health portal [12].

### Data Collection

Data were extracted from electronic health records through a collaborative process involving health technology assessment experts, clinical staff, and data specialists. Two meetings were held to define the data extraction specifications, including variables and inclusion criteria, and review a preliminary dataset for quality and completeness. Extracted variables included test date (year and month), test type (clinical vs self-test), and test result (positive or negative). Months with incomplete data or overlapping phases were excluded to ensure valid comparisons. The data extract contained only the overall test result across the entire dataset. Consequently, the analysis did not distinguish between chlamydia and gonorrhea.

Staff resource use data were collected from the Department of Dermatology and Allergy Centre detailing the number of hours that physicians, nurses, and administrative staff dedicated in a week to working in the STI testing pathway. Staff time included all activities directly related to testing, including patient guidance where required; specimen handling; communication of test results; and follow-up of patients with positive test results, including treatment when indicated. Patients with more complex health care needs requiring additional clinical assessment or management were not included as the study population comprised only individuals who completed testing using the STT. Patients with more complex health care needs were managed through the standard clinical pathway and were therefore outside the scope of the resource analysis. Weekly staff hours were converted into annual full-time equivalents (FTEs) and associated personnel costs based on average gross salaries. Material costs per test (including the self-test kit and other consumables) and laboratory analysis costs were also included. Annual personnel costs were calculated using the annual salaries for each professional group (DKK 986,243 for a senior physician, DKK 499,610 for a nurse, and DKK 489,036 for a medical secretary). All costs are reported in 2025 DKK. For international reference, equivalent values in US dollars can be obtained using the historical exchange rate of July 1, 2025 (DKK 6.3651=US $1).

### Data Analysis

Descriptive statistics were used to summarize testing volume, test frequency, test results, and resource use across the study periods. Because the implementation phases differed in duration, testing activity was summarized both as total number of tests and as the mean monthly number of tests, reported with SDs and minimum and maximum values.

Test frequency was assessed at the patient level within a 1-year observation period. To ensure comparability across implementation phases of differing duration, test frequency was evaluated within calendar years rather than across the duration of each phase. Patients were categorized according to the number of STI tests performed (1 test, 2‐5 tests, and >5 tests), and the distribution of these categories was compared between the pre- and postimplementation periods using the Pearson chi-square test.

Test positivity was calculated as the proportion of positive tests among all tests for chlamydia and gonorrhea. Overall positivity rates were compared across the 3 implementation phases using the Pearson chi-square test. To explore differences by testing modality, positivity rates were also described separately for clinic-based testing and self-testing.

Resource use was assessed as weekly staff time (hours per week) for physicians, nurses, and administrative staff. On the basis of these estimates, FTEs, annual personnel costs, monthly personnel costs, total monthly costs, and costs per test were calculated using the assumptions described above.

Statistical significance was defined as *P*<.05. Analyses were conducted using Stata (version 17; StataCorp).

### Ethical Considerations

In Denmark, register studies do not require formal approval from ethics committees according to legislation [13]. This study was approved by the management of the Department of Dermatology and Allergy Centre and by the Danish Data Protection Agency (journal number 22/30101). Furthermore, all data were available in an anonymized format so that specific individuals could not be identified.

## Results

### Testing Volume

During the defined period (February 19, 2022‐October 20, 2025), a total of 10,250 STI tests were conducted. These tests comprised both clinic-based testing and self-testing and were distributed across the 3 implementation phases shown in Table 1. As the implementation phases differed in duration, direct comparison of the total number of tests across phases should be interpreted with caution. However, Table 1 provides an overview of the distribution of clinic-based testing and self-testing across the study periods and illustrates that the proportion of self-tests relative to clinic-based tests was higher in phase 2 following the introduction of drop-in self-testing.

**Table 1.** Number of tests conducted by test modality and implementation phase.

Periods	Clinic-based testing (n=5034), n (%)	Self-test (n=5216), n (%)	Total (n=10,250), n (%)	Tests per month, mean (SD; range)	Number of months
Phase 0 (preimplementation phase)	1144 (22.7)	—^a^	1144 (11.2)	163 (51; 117‐273)	7
Phase 1 (self-test with booking)	2815 (55.9)	2602 (49.9)	5417 (52.8)	246 (42; 175‐344)	22
Phase 2 (drop-in self-test)	1075 (21.4)	2614 (50.1)	3689 (36)	283 (78; 197‐433)	13

a Not applicable.

**Figure 2.**
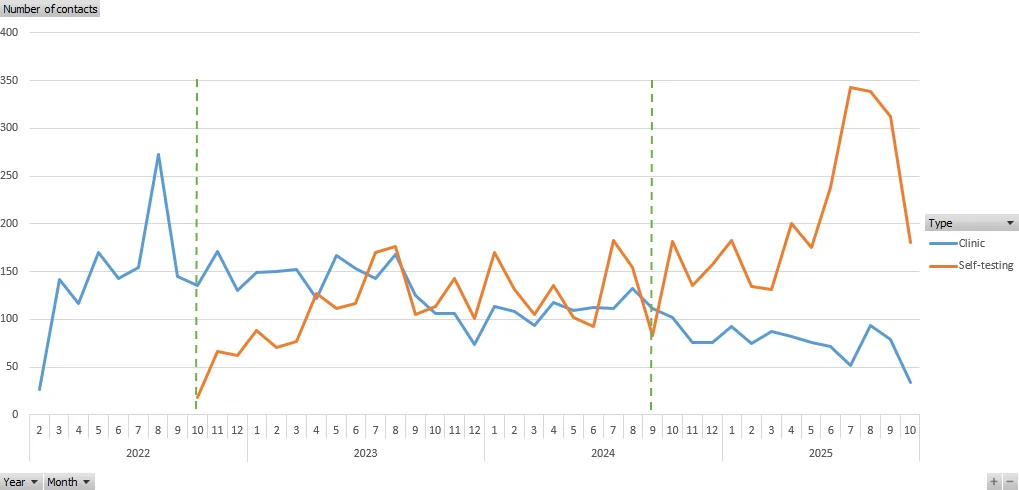
Number of sexually transmitted infection tests conducted in the clinic and via the self-test technology. The vertical markers identify changes from phase 0 to phase 2.

The number of tests performed throughout the study is illustrated in Figure 2. The figure shows the monthly number of tests conducted in the clinic and using the STT. It demonstrates a declining trend in clinic-based testing over the study period (blue line), whereas the number of tests conducted using the STT increased. The green dashed vertical lines mark the beginning of phase 1 and phase 2.

Prior to the implementation of the STT, a mean of 163 (SD 51) tests were conducted per month. Following the introduction of the STT (phase 1), testing activity increased to a mean of 246 (SD 42) tests per month and further to 283 (SD 78) tests per month in phase 2 (drop-in; Table 1).

### Test Frequency

Test frequency was compared between the pre- and postimplementation periods using calendar year–based data. Patients were categorized according to the number of STI tests performed (1 test, 2‐5 tests, and >5 tests). The distribution of patients across these test frequency categories changed after the introduction of the STT (Table 2). A chi-square test demonstrated a statistically significant difference between the pre- and postimplementation periods (*P*=.001), indicating that the distribution of patients across these categories was no longer the same. Prior to implementation, 82.8% (813/982) of patients were tested only once, 16.8% (165/982) were tested 2 to 5 times, and 0.4% (4/982) were tested more than 5 times. After implementation, the corresponding proportions were 77.3% (5466/7068), 22.2% (1569/7068), and 0.5% (33/7068), respectively. The observed statistical difference is primarily driven by a decrease in the proportion of patients tested only once and an increase in those tested 2 to 5 times. The group with more than 5 tests remained very small and changed only marginally, thus contributing minimally to the chi-square result.

**Table 2.** Distribution of patients according to the number of sexually transmitted infection tests performed within a 1-year observation period before and after the introduction of the self-test technology (STT).^a^

Frequency	Before^b^ (n=982), n (%)	After (n=7068), n (%)	Total (n=8050), n (%)
1 test	813 (82.8)	5466 (77.3)	6279 (78)
2‐5 tests	165 (16.8)	1569 (22.2)	1734 (21.5)
>5 tests	4 (0.4)	33 (0.5)	37 (0.5)

a Pearson *χ*2_2_=15.0; *P*=.001.

b “Before” includes only patients tested in the clinic, whereas “after” includes patients tested both in the clinic and via the STT*.*

### Test Results

Table 3 shows the overall distribution of positive and negative results across the 3 implementation phases irrespective of test modality. The purpose of this analysis was to assess whether the overall positivity rate changed following implementation of the STT. The overall positivity rate was 12.6% (1296/10,250). The proportion of positive tests decreased from 15.7% (180/1144) prior to implementation to 12% (652/5417) in phase 1 and 12.6% (464/3689) in phase 2. A Pearson chi-square test demonstrated a statistically significant difference in the overall distribution of positive and negative test results across the 3 implementation phases (*χ*^2^_2_=11.7; *P*=.003).

**Table 3.** Distribution of test results and proportion of positive tests by implementation phase.

Periods	Negative, n (%)	Positive, n (%)
Preimplementation phase (n=1144)	964 (84.3)	180 (15.7)
Phase 1 (self-test with booking implemented; n=5417)	4765 (88)	652 (12)
Phase 2 (drop-in self-test implemented; n=3689)	3225 (87.4)	464 (12.6)
Total (n=10,250)	8954 (87.4)	1296 (12.6)

To further explore whether this overall pattern differed by testing modality, positivity rates were examined separately for clinic-based testing and self-testing (Table 4). Prior to implementation, the positivity rate for clinic-based testing was 15.7% (180/1144). In phase 1, the overall positivity rate decreased to 12% (652/5417), with a lower positivity rate observed for self-testing (243/2602, 9.3%) than for clinic-based testing (409/2815, 14.5%). In phase 2, the overall positivity rate increased slightly to 12.6% (464/3689), with similar positivity rates for clinic-based testing (135/1075, 12.6%) and self-testing (329/2614, 12.6%). Notably, positivity rates for clinic-based testing decreased throughout the study period, whereas positivity rates for self-testing increased from phase 1 to phase 2.

**Table 4.** Positivity rates by implementation phase and testing modality.

Periods and testing modality	Positive tests, n/N (%; 95% CI)
Preimplementation phase	
Clinic-based testing	180/1144 (15.7; 13.7-18.0)
Phase 1	
Clinic-based testing	409/2815 (14.5; 13.3-15.9)
Self-testing	243/2602 (9.3; 8.9-10.5)
Phase 2	
Clinic-based testing	135/1075 (12.6; 10.7-14.7)
Self-testing	329/2614 (12.6; 11.4-13.9)

### Personnel Costs and Resource Use

Table 5 shows an overview of testing activity, staff resource use, and associated costs across the 3 implementation periods. Total weekly staff time decreased from 102 hours prior to implementation to 63.75 hours in phase 2. Reductions were observed across all staff groups, most prominently among physicians (36%) and administrative staff (88%). This reflects that administrative tasks and physician-led clinical activities were increasingly handled automatically or performed by patients themselves through the STT.

Resource use was converted into annual costs based on the assumptions described in the Methods section. Prior to the implementation of self-testing, testing activities corresponded to a total of 3.45 FTEs distributed across professional groups as follows: approximately 0.74 FTEs for physicians, 2.03 FTEs for nurses, and 0.68 FTEs for administrative staff. This resulted in a total annual personnel expenditure of DKK 2,077,177.

Following the introduction of self-testing with appointment booking (phase 1), personnel usee declined to 2.57 FTEs, comprising approximately 0.61 FTEs for physicians, 1.88 FTEs for nurses, and 0.09 FTEs for administrative staff. Annual personnel costs were reduced to approximately DKK 1,576,966, corresponding to a cost reduction of approximately 24% compared with the period prior to the implementation of the STT.

In the current operational model with drop-in self-testing (phase 2), total personnel use amounted to 2.16 FTEs, including approximately 0.47 FTEs for physicians, 1.60 FTEs for nurses, and 0.09 FTEs for administrative staff. This resulted in a total annual personnel cost of approximately DKK 1,305,088, corresponding to a reduction of approximately 37% compared with the period prior to the implementation of the STT.

Using data on analytical costs and expenditures for test kits together with the average number of tests per month reported in Table 1, monthly costs and costs per test were estimated (Table 5). Prior to the implementation of self-testing, an average of 163 (SD 51) tests were performed per month. Total monthly personnel costs amounted to approximately DKK 173,098, whereas consumables and laboratory analyses accounted for approximately DKK 19,027. Overall, this resulted in a total monthly cost of approximately DKK 192,125, corresponding to an estimated cost of DKK 1179 per test.

With the introduction of self-testing with appointment booking (phase 1), test volume increased to an average of 246 (SD 42) tests per month.

**Table 5.** Activity, resource use, and costs across the 3 implementation periods. Values are presented as monthly or annual estimates as indicated. Full-time equivalents (FTEs) include physicians, nurses, and administrative staff. Annual personnel costs were calculated using profession-specific salary levels.

Variables	Before self-testing	Phase 1 (self-testing with booking)	Phase 2 (drop-in self-testing)
Activity			
Tests per month, mean (SD)	163 (51)	246 (42)	283 (78)
Resource use (h per wk)			
Physicians	22.0	18.0	14.0
Nurses	60.0	55.5	47.25
Administrative staff	20.0	2.5	2.5
Total resource use (h per wk)	102.0	76.0	63.75
FTEs	3.45	2.57	2.16
Personnel costs (DKK; DKK 6.3651=US $1 as of July 1, 2025)			
Annual personnel costs	2,077,177	1,576,966	1,305,088
Monthly personnel costs	173,098	131,414	108,757
Other costs (DKK per month)			
Materials and laboratory costs	19,027	28,716	33,035
Total costs (DKK per month)	192,125	160,130	141,792
Cost per test (DKK)	1179	651	501

## Discussion

### Principal Findings

This evaluation examined the operational, clinical, and economic consequences of implementing an STT for the diagnosis of STIs at the Department of Dermatology and Allergy Centre, Odense University Hospital, and demonstrated that the implementation of the STT increased testing activity while reducing personnel costs and lowering the cost per test. The intervention was introduced in two phases: (1) as appointment-based self-testing (phase 1) and (2) as a drop-in solution (phase 2), allowing for assessment of both gradual and fully decentralized implementation.

Across the study period, a substantial increase in overall testing activity was observed, with mean monthly test volume rising from 163 (SD 51) prior to implementation to 246 (SD 42) in phase 1 and 283 (SD 78) in phase 2. Overall, this indicates a clear increase in testing activity after the introduction of the STT and again following the transition to a drop-in model, which allowed patients to attend without prior appointment or contact with a health care professional. This pattern is consistent with the World Health Organization’s self-care intervention framework, which conceptualizes self-testing as a means of lowering structural, temporal, and psychological barriers to care by transferring parts of the diagnostic pathway from the health care system to the individual [14]. By reducing reliance on appointment scheduling, clinical encounters, and direct staff involvement, self-testing enhances accessibility and convenience, which is reflected in the increased uptake observed in this study.

The shift from clinic-based testing to self-testing after the introduction of the drop-in model suggests that self-testing became integrated as a primary testing modality rather than a supplementary service. From an implementation perspective, this development aligns with normalization process theory, which emphasizes how new practices become embedded in routine care once they are coherent, manageable, and perceived as beneficial by both users and staff. The transition from appointment-based to drop-in self-testing appears to have facilitated such normalization [15].

The analysis demonstrated a shift in patient testing patterns, with a decrease in individuals tested only once and an increase in those tested 2 to 5 times. This change can be interpreted using the behavioral model of health service use by Andersen [16], where improved enabling factors such as ease of access, reduced waiting time, and lower perceived stigma lead to increased use independent of changes in clinical need. Thus, self-testing may have reduced practical and psychological barriers to repeat testing, thereby facilitating more regular engagement with STI screening among users. However, the proportion of individuals being tested more than 5 times remained small and changed only marginally across phases. This suggests that expanded access through self-testing did not result in widespread overuse; instead, the findings indicate a targeted behavior change primarily among individuals who benefited from improved access to repeated testing. Thus, self-testing is a way for people to take responsibility for their own health and places a responsibility to integrate people into existing health care systems, allowing people to make a well-informed decision [17]. This is consistent with the qualitative evaluation of the STT that showed that patients were more satisfied with using the STT rather than attending face-to-face consultations and that using the STT facilitated self-care [10].

The overall positivity rate declined from 15.7% (180/1144) prior to implementation to between 12% (652/5417) and 12.6% (464/3689) during the self-testing phases. This was higher than the national positivity rate for chlamydia in Denmark in 2024 (8.5%), which was published in 2025 [18]. This indicates that the technological solution is an attractive option for individuals who might otherwise not undergo testing and, therefore, has the potential to increase testing uptake. From a screening perspective, this reduction is a well-recognized phenomenon often referred to as risk dilution: when barriers to testing are lowered, individuals at lower average risk are more likely to participate, which reduces the overall positivity rate even if absolute case detection increases [19]. Thus, the observed decline should not be interpreted as reduced clinical relevance or effectiveness of testing. Furthermore, in phase 2, the positivity rate among self-tests increased to a level comparable to that of clinic-based testing. This suggests that, over time, self-testing attracted a patient population with a risk profile similar to that of those traditionally tested in outpatient clinics. From an implementation perspective, this may reflect a stabilization phase in which early exploratory use is replaced by sustained, need-driven use, consistent with normalization process theory [15].

The implementation of the STT had substantial organizational implications. Total weekly personnel time decreased from 102 hours prior to self-testing to 63.75 hours in phase 2. The largest reduction was observed among administrative staff, whose workload was previously driven by appointment booking, telephone contact, and coordination. This pattern is consistent with task shifting and task automation frameworks in which administrative and procedural tasks are either automated or transferred to patients themselves [20]. Reductions were also observed among physicians and nurses, reflecting decreased need for clinician involvement in routine, asymptomatic STI testing. Hence, from a task shifting perspective, self-testing can be understood as a form of patient-mediated task redistribution in which parts of the diagnostic process are safely delegated to users with minimal loss of quality and substantial gains in efficiency. It should be noted that examining potential variation in the weekly number of hours allocated to testing activities over a longer period would be relevant as such variation may influence annual personnel costs.

Despite increased test volume, total monthly costs decreased markedly following implementation, driven primarily by reductions in personnel expenses. Consequently, the cost per test declined from approximately DKK 1179 before self-testing to DKK 501 in phase 2. This finding reflects basic health economic principles, particularly substitution of semifixed personnel costs with variable material and laboratory costs. Moreover, the results are consistent with the lowest effective cost level principle as testing tasks were redistributed to align with the lowest appropriate level of competence, enabling more efficient use of health care personnel resources [21]. As testing volumes increase, average costs per test fall, resulting in improved cost efficiency.

These findings are consistent with those of prior economic evaluations of STI self-testing, which have demonstrated that reductions in staff time and clinic resource use can outweigh increased laboratory expenditures, leading to net cost savings [22]. However, the findings of Wilson et al [22] are based on the principles of pooling NAATs during laboratory analysis, whereas our study used NAATs with laboratory diagnosis of each test. There is little knowledge on using NAATs and STTs in an STI clinical setting and, therefore, little to compare our findings with regarding cost-effectiveness. Thus, the present study adds real-world evidence from a hospital-based setting, reinforcing the economic viability of self-testing as a scalable diagnostic strategy.

### Limitations and Implications for Future Research

Several limitations must be considered. This study used an observational before-and-after design based on aggregated electronic patient record data, which precludes causal attribution. Changes in testing behaviors, positivity rates, and volume may also have been influenced by external factors such as seasonal variation, shifts in population behaviors, or broader public health trends.

Personnel resource use was estimated rather than measured through direct time tracking, introducing potential uncertainty. Although estimates were developed by staff with detailed knowledge of workflows, some degree of over- or underestimation is possible. Additionally, reliance on routinely collected data carries an inherent risk of registration errors that cannot be fully identified in aggregated analyses.

Future research should include longer follow-up periods to assess whether testing volume, testing frequency, and positivity rates stabilize over time, as well as more granular data on patient characteristics and risk profiles. Systematic time registration would further strengthen economic evaluations and support more precise estimates of resource redistribution.

### Conclusions

This evaluation and analysis of activity and resource data demonstrates that the implementation of the STT increased testing activity while reducing personnel costs and lowering the cost per test. Despite methodological limitations, the findings indicate that self-testing represents an effective and resource-efficient solution capable of accommodating higher testing volumes without requiring additional staff resources.

## Abbreviations

FTE: full-time equivalent
NAAT: nucleic acid amplification test
STI: sexually transmitted infection
STT: self-test technology
